# *Lactiplantibacillus plantarum* GUANKE Enhances Antiviral Defense Against Respiratory Syncytial Virus Through the STING-TBK1-IRF3-IFN Pathway

**DOI:** 10.3390/nu18030399

**Published:** 2026-01-26

**Authors:** Kun Yue, Simin Lu, Hanyu Ma, Jielan Mi, Qianjin Fan, Tao Yang, Yuanming Huang, Liqiong Song, Zhihong Ren, Lili Ren, Jianguo Xu

**Affiliations:** 1 Research Unite for Unknown Microbe, Chinese Academy of Medical Sciences & Peking Union Medical College, Beijing 100730, China; 2 Institute of Pathogen Biology, Chinese Academy of Medical Sciences & Peking Union Medical College, Beijing 100730, China; 3 National Key Laboratory of Intelligent Tracking and Forecasting for Infectious Diseases, National Institute for Communicable Disease Control and Prevention, Chinese Center for Disease Control and Prevention, Beijing 102200, China; 4 Basic Theory for Chinese Medicine, China Academy of Chinese Medical Sciences, Beijing 100700, China; 5 Institute of Public Health, Nankai University, Tianjin 300071, China

**Keywords:** probiotics, *Lactiplantibacillus plantarum* GUANKE, respiratory syncytial virus, interferon

## Abstract

**Background/Objectives:** To investigate the antagonistic effect of probiotic *Lactiplantibacillus plantarum* GUANKE against respiratory syncytial virus (RSV) and its underlying molecular mechanisms. **Methods:** in vitro cell models (A549 and HEp2 cells) and an in vivo mouse model (BALB/c mice) were employed. RT-qPCR, TCID50 assay, immunofluorescence, ELISA, Western blot, and histopathological analysis were used to investigate the effects of GUANKE on RSV replication, inflammatory responses, and the type I interferon pathway. **Results:** Oral administration of GUANKE effectively cleared RSV and alleviated RSV-induced pulmonary inflammatory responses. GUANKE inhibited viral replication. The GUANKE intervention group exhibited significantly reduced pathological damage to lung tissue and decreased the expression of inflammatory cytokines (IL-1β, IL-6, MCP-1, TNF-α). GUANKE augmented the early type I interferon response and activated the STING-TBK1-IRF3-IFN signaling pathway. **Conclusions:** GUANKE exerts anti-RSV effects by enhancing the early type I interferon response and activating the STING-TBK1-IRF3-IFN signaling pathway, thereby inhibiting RSV replication and alleviating pulmonary inflammatory responses. This suggests its potential value as an anti-RSV agent.

## 1. Introduction

Respiratory syncytial virus (RSV) is a significant viral pathogen that predominantly affects infants and young children with underdeveloped immune systems [1,2] and immunocompromised older adults [3,4], constituting a primary causative agent of respiratory tract infections on a global scale [5]. RSV may also cause lower respiratory tract diseases and long-term respiratory complications, such as capillary bronchitis, pneumonia, and asthma [6,7]. As reported by the World Health Organization (WHO), RSV stands as the primary factor leading to the hospitalization of millions of children across the globe annually. This is particularly prominent in low- and middle-income countries, where the rates of RSV-related hospitalization and mortality are notably higher compared to those in other areas [2,8]. Currently, therapeutic drugs against RSV (e.g., ribavirin) have significant efficacy limitations and resistance issues, and are accompanied by adverse effects [9]. Meanwhile, multiple RSV vaccines (e.g., GSK Arexvy, Pfizer Abrysvo, Moderna mResvia) have been approved for adults aged 60 and above. Abrysvo is also licensed for maternal vaccination to protect infants. The development of RSV vaccines has faced numerous challenges in terms of long-term protective efficacy, safety, and broad-spectrum protection against different viral strains [10]. Consequently, there is an urgent and unmet need for the development of novel, safe, and efficacious therapeutic strategies against RSV.

Probiotics have recently garnered significant attention as a potential therapeutic strategy. A substantial body of research has demonstrated that the administration of particular probiotic strains can protect respiratory tract infections, as evidenced by clinical trials and preclinical models [11,12,13,14,15,16,17]. Probiotics are alive microorganisms that are beneficial to the host health [18]. Probiotics have been demonstrated to combat respiratory viral infections through a variety of mechanisms, including improvement of mucosal barrier function, inhibition of viral replication, secretion of metabolites with antiviral activity, and stimulation of host innate and adaptive immunity. For instance, *Enterococcus faecium* NCIMB 10415 has been observed to impede influenza virus replication through direct interaction with the viral surface [19]. An extracellular polysaccharide derived from *Lactobacillus delbrueckii* ssp. *bulgaricus* OLL1073R-1 has been proven to exert inhibitory effects on influenza virus infection and enhance barrier damage [20]. The results demonstrated that *Lactiplantibacillus* pentosus CCFM1227 exhibited the capacity to inhibit viral replication and attenuate lung pathology by producing Desaminotyrosine and increasing IFN-β [21]. Mechanistic studies have demonstrated that probiotics can exert protective effects through pathways that enhance intrinsic immune function. For instance, the oral administration of probiotic L-92 has been shown to enhance natural killer cell activity and increase various antiviral cytokines and chemokines, thereby protecting against viral infections [22]. *Lactobacillus johnsonii* (*L. johnsonii*) has been observed to attenuate respiratory viral infections through metabolic reprogramming and immune cell regulation [23]. Furthermore, *Lactobacillus gasseri* SBT2055 treatment has been demonstrated to stimulate the expression of antiviral genes Mx1 and Oas1a [24]. Furthermore, probiotics have been shown to mitigate the deleterious effects of respiratory infections by modulating the inflammatory response. For instance, *L. johnsonii* supplementation significantly reduced airway hyperresponsiveness and decreased inflammation and mucus-producing goblet cells [25].

A multitude of studies have demonstrated the protective effect of *Lactiplantibacillus plantarum* against respiratory viral infections, including influenza virus and neocoronavirus [26,27,28,29,30,31]. However, majority of studies focused on probiotic mixtures containing *Lactiplantibacillus plantarum*. A probiotic mixture comprising *Lactobacillus rhamnosus* GG, *Escherichia coli* Nissle 1917, and VSL#3 (provided by VSL Pharmaceutical Company, containing *Streptococcus thermophilus*, *Bifidobacterium breve*, *Bifidobacterium longum*, *Bifidobacterium infantis*, *Lactobacillus acidophilus*, *Lactobacillus plantarum*, *Lactobacillus paracasei* and *Lactobacillus delbrueckii subspecies*; note that the formula of VSL#3 has been altered since 2016 following a change in product ownership) has been demonstrated to exhibit resistance to RSV infection by promoting IFN-β production by alveolar macrophages. Furthermore, the probiotic mixture reversed intestinal and lung flora dysbiosis and increased the level of short-chain fatty acid in RSV-infected mice [32]. In another study, *Lacticaseibacillus casei* AMBR2, *Lacticaseibacillus rhamnosus* GG, and *Lactiplantibacillus plantarum* WCFS1 were formulated as a live biotherapeutic throat spray, which could significantly reduce the cytopathic effects of respiratory viruses, including RSV [33]. Despite these studies above mentioned suggest that specific probiotic strains and their metabolites may attenuate RSV infection by inhibiting viral protein expression [34], re-balancing microbial flora [32], and modulating host immune responses [35,36,37] and metabolism [38], the underlying mechanism through which probiotics affect early antiviral immune response against RSV remains to be fully elucidated.

Our previous work found that *Lactiplantibacillus plantarum* GUANKE is a probiotic strain with an excellent safety profile [39] and immunomodulatory ability [40], which can combat influenza virus via dendritic cells [41] as well as mitigate the inflammatory response of lung tissue caused by SARS-CoV-2 [42]. The objective of this study was to investigate the specific molecular mechanism of antiviral activity of GUANKE. To examine GUANKE’s paracrine effects and exclude direct bacterial contact, we used a 0.4 μm pore transwell system to separate probiotics from A549 cells; this pore size permits only soluble factor diffusion, while preventing probiotic translocation. Initially, we confirmed the substantial inhibitory effect of GUANKE on RSV replication in both in vitro model and in vivo model in the present work. Additionally, we observed the effect of GUANKE on enhancing the early type I interferon response. The finding of the activating STING-TBK1-IRF3-IFN pathway by GUANKE in response to RSV infection provides a new theoretical basis and experimental support for developing probiotic-based intervention approaches targeting respiratory viral infections.

## 2. Methods and Materials

### 2.1. Bacterial Culture

The *Lactiplantibacillus plantarum* GUANKE strain, which was isolated from the fecal samples of healthy individuals, has been deposited in the China General Microbiological Culture Collection Center (CGMCC) under the accession number CGMCC NO.21720. The GUANKE strain was cultured in Man-Rogosa-Sharpe (MRS) medium at 37 °C in a 5% CO_2_ incubator. For the initial culture process, the preserved strain was first inoculated onto solid MRS medium. After that, a single colony was selected and transferred to liquid MRS medium for cultivation. Bacterial cells in the logarithmic growth phase were harvested and centrifuged at 4000× *g* for 10 min. The collected bacteria were rinsed twice with phosphate-buffered saline (PBS) and finally resuspended in PBS to prepare for gavage administration.

### 2.2. Virus Culture

The RSV-A2 have been maintained by Institute of Pathogen Biology and grown in human laryngeal epithelial (HEp-2) cells. Viral titers were quantified and expressed as the 50% tissue culture infectious dose (TCID_50_).

### 2.3. Mouse Model

Four- to six-week-old male BALB/c mice, obtained from Beijing Vital River Laboratory Animal Technology Co., Ltd. (Beijing, China), were housed in individually ventilated cages (IVCs) within a specific pathogen-free (SPF) environment. To evaluate the effect of oral administration of GUANKE on improving RSV-infected mice, the mice were randomly divided into three groups (6 or 7 mice/group): non-infected group, RSV-infected PBS-treated group and RSV-infected GUANKE-treated group. The mice were treated with a mixture of antibiotics for three days to deplete their gut microbiota. The antibiotic cocktail consisted of ampicillin (1 g/L), vancomycin hydrochloride (0.5 g/L), gentamicin sulfate (0.5 g/L), amphotericin B (0.1 g/L), and metronidazole (8 g/L). Subsequently, the mice in each group were intervened for five days. The mice in the Control group, RSV + PBS group, and RSV + GUANKE group were orally administered PBS (200 μL), PBS (200 μL), or GUANKE (2 × 10^9^ CFU/mouse, 200 μL), respectively. Each mouse was first weighed, then anesthetized through intraperitoneal injection of pentobarbital, and subsequently subjected to intranasal infection with 1 × 10^6^ plaque-forming units (PFU) of RSV. The body weight of the mice was measured and recorded daily throughout the experiment. Four days after infection, the rectal temperature of each mouse was measured and recorded using a mouse thermometer (FT3400) prior to specimen collection, followed by lung specimen collection and examination.

### 2.4. In Vitro Co-Culture Cell Model Establishment

To avoid direct contact between probiotics and host cells, as previously described in studies [43,44], a specialized co-culture model was established using a Transwell system (Corning, NY, USA) in 12-well plates. This model comprised two layers: A549 cells (including wild-type A549 cells, STING1 Knockout A549 cells, and IFNAR1 Knockout A549 cells) in the apical chamber and GUANKE cells in the basolateral chamber. The STING1 Knockout A549 cell line (YKO-H503) and IFNAR1 Knockout A549 cell line (YKO-H503) were purchased from Ubigene (Guangzhou, China). All cells were maintained in DMEM medium supplemented with 10% fetal bovine serum (FBS), in a humidified incubator at 37 °C with 5% CO_2_.

For the co-culture setup, Transwell inserts with a permeable membrane (pore size: 0.4 μm) were placed into the wells of the 12-well plates. A549 cells were seeded at a density of 3 × 10^5^ cells/well on the apical side of the Transwell membrane and allowed to adhere overnight. Subsequently, GUANKE cells were added to the basolateral chamber. This configuration established two separate cellular compartments that could communicate via soluble factors diffusing across the membrane, without direct cell-to-cell contact, thereby simulating paracrine interactions within the gut–lung axis.

Sample collection at specific time points post-infection: A549 cells were collected for Western blot analysis to evaluate protein expression levels at 8 h post-infection with RSV. Total RNA was extracted from A549 cells for RT-qPCR to detect gene expression levels at 24 h post-infection with RSV. Cell culture supernatants were collected for subsequent ELISA to quantify the expression and secretion of the cytokine IFN-β at 48 h or longer post-infection with RSV.

### 2.5. Immunofluorescence

HEp-2 cells adherent on glass coverslips were pretreated with GUANKE for 24 h, followed by RSV infection for 48 h. Cells were fixed with 4% paraformaldehyde (PFA), permeabilized with 0.05% Triton X-100, blocked with 3% BSA, and incubated overnight with anti-RSV F primary antibody (1:200; Sino Biological, Beijing, China). After staining with fluorophore-conjugated secondary antibody (Alexa Fluor 488, 1:500; ZSGB-BIO, Beijing, China) and DAPI (Solarbio, Beijing, China), coverslips were mounted onto slides using ProLong Gold Antifade Mountant (Solarbio) and stored at 4 °C in the dark. Fluorescence images were acquired using a confocal laser scanning microscope. Emission filters were set to 488 nm (green) and 405 nm (DAPI). Image quantification was performed using ImageJ version 1.53e.

### 2.6. TCID_50_ Assay

Viral titers were determined by TCID50 assay using the Spearman-Kärber algorithm. Serially diluted virus samples were inoculated into HEp-2 cell monolayers (2 × 10^4^ cells/well) in 96-well plates. After 1 h adsorption, cells were maintained in RPMI-1640 with 2% FBS. Following 72 h incubation, RSV-induced syncytia formation was evaluated by immunofluorescence.

### 2.7. Histopathological Analysis

Fresh lung tissues were harvested and immobilized in 4% formalin-buffered solution for a period exceeding 24 h. Subsequently, the tissue samples were sent to Wuhan Servicebio Technology Co., Ltd. (Wuhan, China). for paraffin embedding, sectioning, and subsequent staining processes. Hematoxylin and eosin (H&E) staining was performed on the paraffin-embedded sections, after which the pathological changes in the lung tissues were observed under a light microscope. Four key pathological parameters in lung sections: alveolitis, interstitial inflammation, perivascularitis, and peribronchiolitis. Each parameter was scored independently on an ordinal scale from 0 to 4, where 0 indicates normal lung histology (no detectable lesions) and 4 indicates severe pathology [45,46]. The overall lung pathology score was calculated as the sum of the individual parameter scores.

### 2.8. Quantitative Real-Time PCR (qRT-PCR)

The mRNA expression level was determined via real-time quantitative polymerase chain reaction (qRT-PCR). Total RNA was isolated from tissue samples using RNAiso Plus (Trizol reagent, TaKaRa, Kusatsu, Shiga, Japan), and then reverse-transcribed into complementary DNA (cDNA) with the FastKing gDNA Dispelling RT SuperMix kit (Tiangen Biotech, Beijing, China). For the detection of gene transcriptional expression, the Bioer Fast 9600 instrument (Bioer Technology, Hangzhou, China) was employed, following the protocol provided by the SuperReal PreMix Pix Plus kit (Tianen Biotechnology).

The qRT-PCR amplification was carried out under the following thermal cycling conditions: an initial denaturation step of 95 °C for 10 min (1 cycle); followed by 40 cycles of denaturation at 95 °C for 15 s, annealing at 55 °C for 20 s, and extension at 72 °C for 20 s; and a final melting curve analysis consisting of 95 °C for 10 s, 65 °C for 1 min, and 97 °C for 1 s (1 cycle). The primers utilized for PCR amplification are listed in Table 1.

The 2^−ΔΔCT^ algorithm was applied to normalize the expression level of each target gene relative to the internal reference gene β-actin.

### 2.9. Western Blotting

Protein samples were prepared using RIPA lysis buffer (Solarbio) supplemented with both protease inhibitors and phosphatase inhibitors. To standardize the protein concentration across all samples, the BCA Protein Assay Kit (LABLEAD, Beijing, China) was employed for concentration determination and adjustment. Subsequently, loading buffer was added to each protein sample, followed by boiling at 100 °C for 10 min to denature the proteins. The processed protein samples were separated via 10% sodium dodecyl sulfate-polyacrylamide gel electrophoresis (SDS-PAGE) and then electrophoretically transferred onto a nitrocellulose (NC) membrane (Millipore, Burlington, MA, USA). The membrane was blocked with 5% BSA for 2 h. Membranes were then incubated overnight with primary antibody (STING, phospho-STING, CST; phospho-TBK1, Abcam; TBK1, IRF3, phospho-IRF3, Proteintech, Rosemont, IL, USA) at 4 °C and washed 3 times with TBST for 5 min each. Secondary antibody (Goat anti Rabbit IgG, LABLEAD) was incubated for 1 h at room temperature and washed 3 times with TBST for 5 min each. The bands were visualized using ECL (BOSTER, Pleasanton, CA, USA) with Amersham Imager 680R (General Electric, Boston, MA, USA). The grey values of the bands were calculated and normalized to those of β-actin using Image J.

### 2.10. Enzyme-Linked Immuno Sorbent Assay (ELISA)

ELISA was employed for the quantification of cytokines. Specifically, mouse interleukin-6 (IL-6, Invitrogen, Waltham, MA, USA), interleukin-1β (IL-1β, Invitrogen, Waltham, MA, USA), tumor necrosis factor-α (TNF-α, Invitrogen, Waltham, MA, USA), monocyte chemoattractant protein-1 (MCP-1, R&D, Minneapolis, MN, USA), and mouse interferon-β (IFN-β, R&D) in lung tissue supernatants were measured using corresponding ELISA kits. Additionally, human IFN-β (JONLNBIO, Shanghai, China) levels in the supernatants of cultured cells were determined with the respective ELISA kit, strictly following the manufacturers’ instructions. All absorbance values were read at a wavelength of 450 nm using a microplate reader.

### 2.11. Statistical Analysis

All statistical analyses involved in this study were conducted with GraphPad Prism 8.0 software. For comparisons among multiple groups, one-way analysis of variance (ANOVA) was adopted. When analyzing the statistical significance between two groups, a two-tailed unpaired Student’s *t*-test was applied, and the results are expressed as mean ± standard error of the mean (SEM). A *p* value less than 0.05 was defined as statistically significant, with the following notation: * *p* < 0.05, ** *p* < 0.01, *** *p* < 0.001, **** *p* < 0.0001.

## 3. Results

### 3.1. Lactiplantibacillus plantarum GUANKE Inhibits RSV Replication In Vitro Cell Model

**Figure 1:**
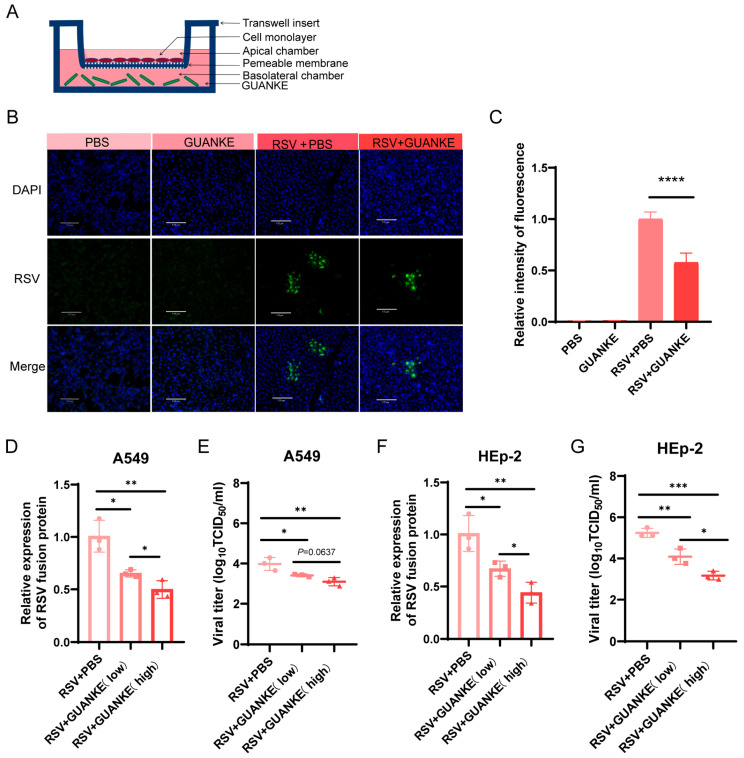
Effects of GUANKE on RSV infection in vitro cell models. (**A**) Schematic diagram of the in vitro cell model system. (**B**) Immunofluorescence detection of RSV at 48 h post-infection (hpi), A549 cells were pretreated with or without GUANKE (MOI = 50) for 24 h and then infected with RSV (MOI = 0.1). Cell nuclei were visualized by DAPI staining and appeared blue fluorescence under the microscope. The RSV F protein was visible green. Scale bars represent 170 μm. (**C**) Quantification of immunofluorescence signal. (**D**) Viral load in A549 cells were pretreated with or without GUANKE (MOI = 20 and MOI = 50) was quantified by RT-qPCR. (**E**) Virus titer in A549 cell supernatants was measured by TCID_50_ assay. (**F**) Viral load in HEp2 cells was quantified by RT-qPCR. (**G**) Virus titer in HEp2 cell supernatants was measured by TCID_50_ assay. Data are presented as mean ± SEM. *p* < 0.05 means the statistical difference. * *p* < 0.05; ** *p* < 0.01; *** *p* < 0.001; **** *p* < 0.0001.

To demonstrate the effect of GUANKE on RSV, we conducted relevant in vitro experiments. Prior to evaluating its antiviral activity, GUANKE (MOI = 50) showed no significant cytotoxicity against A549 and HEp-2 cells (Figure S1). The schematic diagram of the in vitro cell model system is shown in Figure 1A. A549 cells were either pretreated with GUANKE (MOI = 50) for 24 h or left untreated, followed by infection with RSV (MOI = 0.1). Cells were harvested/analyzed at 48 h post-infection (hpi) with RSV. The cell nucleus was stained blue with DAPI, and the RSV F protein was displayed in green (Figure 1B). The quantitative analysis of immunofluorescence signals (Figure 1C) revealed that GUANKE pretreatment significantly reduced the immunofluorescence signals of RSV F protein compared to non- pretreated cells (*p* < 0.01). The viral load in A549 cells, as determined by RT-qPCR (Figure 1D), showed that GUANKE pretreatment markedly decreased the viral load (*p* < 0.001). Similarly, the viral titer in the supernatant of A549 cells, measured by the TCID_50_ method (Figure 1E), was significantly reduced in the GUANKE pretreatment group compared to the PBS pretreatment group (*p* < 0.01). Furthermore, in HEp2 cells, the viral load measured by RT-qPCR (Figure 1F) and the viral titer in the supernatant measured by the TCID50 method (Figure 1G) also exhibited significant reductions in the GUANKE pretreatment group compared to the PBS pretreatment group.

### 3.2. Oral Supplementation of GUANKE Effectively Reduces RSV Infection and Alleviates RSV-Induced Pulmonary Inflammatory Response

**Figure 2:**
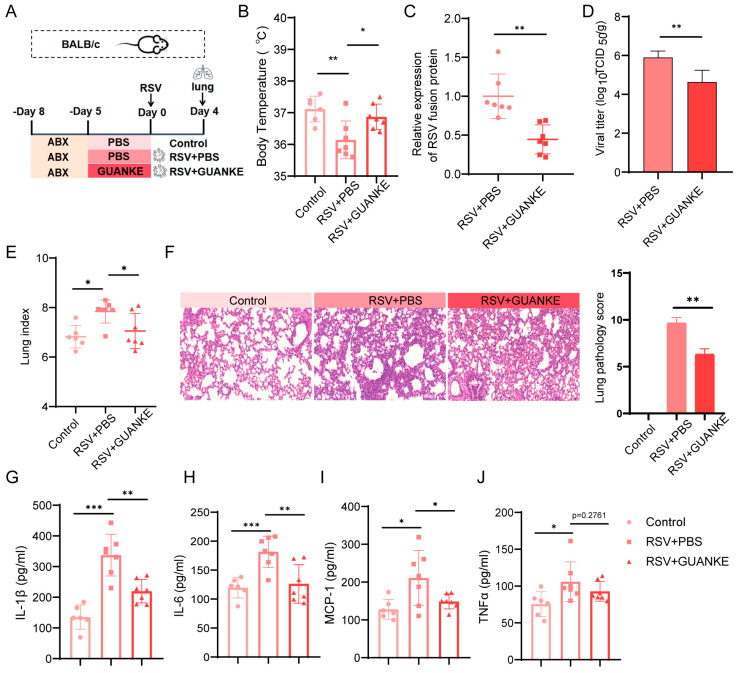
Effects of GUANKE on RSV-infected animal models. (**A**) Animal model experimental design. BALB/c mice received antibiotics (ABX) for 3 days. Then, mice were given PBS or GUANKE. On Day 0, mice were infected with RSV. Lungs were collected on Day 4 post-infection. Groups: Control, RSV + PBS, RSV + GUANKE. (**B**) Body temperature detection of the animal models. (**C**) Viral load in the models was quantified by RT-qPCR. (**D**) The titer of the virus was determined. (**E**) Lung index of each group (lung index = wet lung weight[mg]/body weight[g]). (**F**) HE staining and pathology score of lung tissues of mice. The magnification used was 200×. (**G**–**J**) Contents of the inflammatory factors IL-1β (**G**), IL-6 (**H**), MCP-1 (**I**) and TNF-α (**J**) in mice lungs are determined. Data are presented as mean ± SEM. *p* < 0.05 means the statistical difference. * *p* < 0.05; ** *p* < 0.01; *** *p* < 0.001.

The experimental design of the animal model is presented in Figure 2A. Compared with the control group (37.12 ± 0.1662 °C), the RSV-infected group (36.14 ± 0.2256 °C) showed a significant decrease in body temperature. In contrast, GUANKE intervention (36.87 ± 0.1523 °C) restored body temperature to a level close to that of the control group (Figure 2B). Quantitative analysis of viral load revealed that the oral GUANKE intervention group exhibited a significantly lower viral load than the RSV-infected group (Figure 2C). Consistently, GUANKE intervention also markedly reduced RSV titer (Figure 2D). The lung index (indicative of lung injury) of each group (Figure 2E) also revealed that the lung index of the RSV-infected mice was significantly higher than that of the control group, while oral GUANKE intervention markedly reduced the lung index. HE staining revealed obvious pathological lesions in the lung tissue of the RSV-infected group: increased alveolar dilation, focal mild thickening of alveolar walls with widened septa; slight granulocyte infiltration in the interstitium (including intrapulmonary connective tissue and blood vessels), a small amount of epithelial cells and eosinophilic substances in the bronchiole lumens; scattered macrophages and lymphocytes in alveoli, and occasional focal perivascular lymphocyte infiltration. In contrast, the oral GUANKE intervention group showed significantly alleviated pathological changes mentioned above. Consistent with these morphological observations, semi-quantitative analysis of lung pathology scores demonstrated that the RSV-infected group exhibited a markedly elevated lung pathology score, while oral GUANKE intervention significantly reduced the score (Figure 2F). Furthermore, the levels of inflammatory factors IL-1β (Figure 2G), IL-6 (Figure 2H), MCP-1 (Figure 2I), and TNF-α (Figure 2J) in the mouse lungs were measured. Compared with the control group, the RSV infection group showed markedly elevated levels of these inflammatory factors, while the level of cytokines of IL-1β, IL-6 and MCP-1 reduced significantly in GUANKE intervention group. In summary, oral GUANKE can effectively reduce RSV infection and mitigate RSV-induced pulmonary inflammatory responses.

### 3.3. Lactiplantibacillus plantarum GUANKE Stimulation Upregulates ISG Expression in RSV-Infected Cells

**Figure 3:**
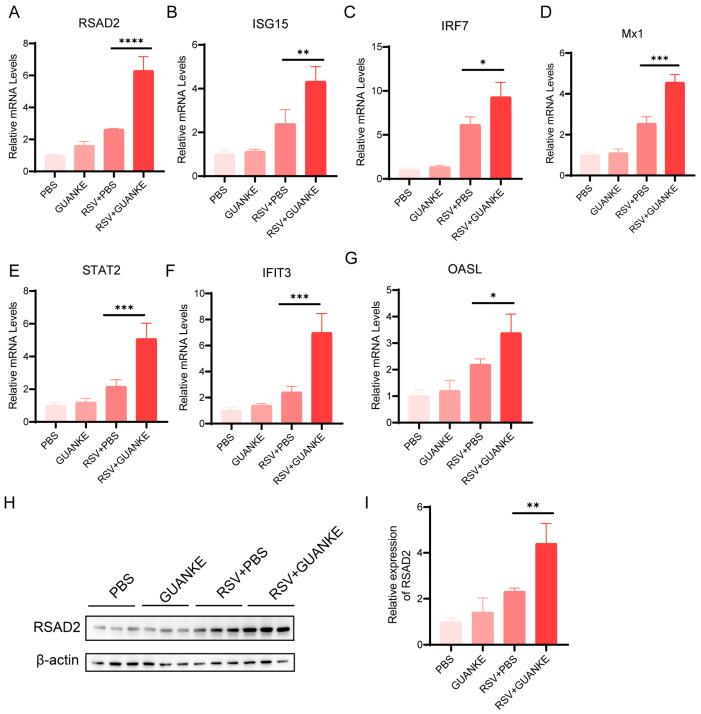
Effects of GUANKE on the expression of antiviral genes and proteins in in vitro cell models. (**A**–**G**) Expression of genes *RSAD2*, *ISG15*, *IRF7*, *MX1*, *STAT2*, *IFIT3* and *OASL* was quantified by RT-qPCR in A549 cells at 48 h post-infection. (**H**) RSAD2 protein expression in A549 cells at 48 hpi was detected by Western blotting. (**I**) Quantification of RSAD2 protein levels normalized to β-actin from Western blot results. Data are presented as mean ± SEM. *p* < 0.05 means the statistical difference. * *p* < 0.05; ** *p* < 0.01; *** *p* < 0.001, **** *p* < 0.0001.

Given the crucial roles of ISG genes in antiviral responses, we assessed the impact of GUANKE on these genes in RSV-infected cell models. As depicted in Figure 3, quantitative analysis via RT-qPCR at 48 h post-infection revealed significant upregulation of *RSAD2* (Figure 3A), *ISG15* (Figure 3B), *IRF7* (Figure 3C), *MX1* (Figure 3D), *STAT2* (Figure 3E), *IFIT3* (Figure 3F), and *OASL* (Figure 3G) genes in A549 cells (*p* = 0.0019, *p* = 0.0229, *p* = 0.0442, *p* = 0.0024, *p* = 0.0081, *p* = 0.0116, *p* = 0.0573 for *RSAD2*, *ISG15*, *IRF7*, *MX1*, *STAT2*, *IFIT3* and *OASL*, respectively). As shown in Figure 3H, Western blot analysis confirmed increased RSAD2 protein expression in GUANKE-treated cells, in consistent with corresponding quantitative data normalized to β-tubulin (Figure 3I). These results indicate that GUANKE can effectively stimulate ISG expression in RSV-infected cells and enhance antiviral defenses.

### 3.4. Lactiplantibacillus plantarum GUANKE Exerts Antiviral Effects via the Type I Interferon Pathway

**Figure 4:**
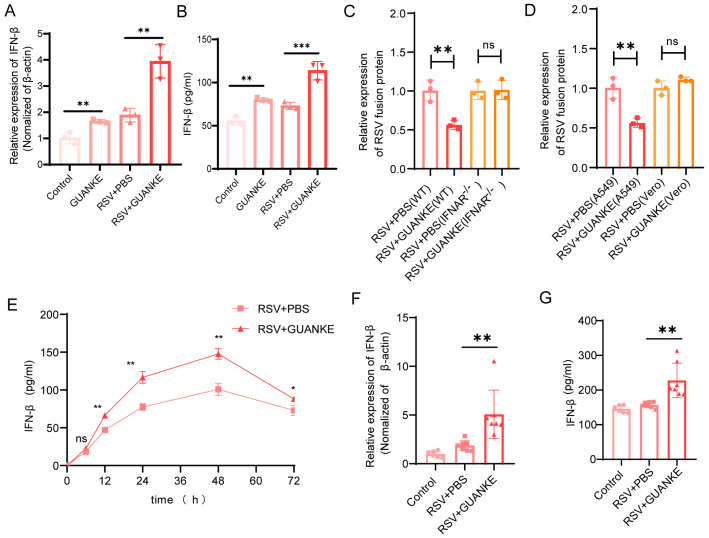
Effects of GUANKE on IFN-β response in vitro and in vivo. (**A**) IFN-β expression in A549 cells was quantified by RT-qPCR. A549 cells were pretreated with or without GUANKE for 24 h and then infected with RSV (MOI = 0.1) for 24 h. (**B**) IFN-β content in A549 cell supernatants was detected by ELISA at 48 h post-infection. A549 cells were pretreated with or without GUANKE for 24 h and then infected with RSV (MOI = 0.1). (**C**,**D**) Viral load in IFNAR^−/−^ cells (**C**) or Vero cells (IFN-deficient cell line) (**D**) was quantified by RT-qPCR. (**E**) Time-course analysis of IFN-β content in A549 cell supernatants detected by ELISA. Supernatants were collected at indicated time points post-infection. (**F**) IFN-β expression in mouse lung tissues was quantified by RT-qPCR at 1 day post-infection (dpi). (**G**) IFN-β content in mouse lung homogenates was detected by ELISA at 1 dpi. Data are presented as mean ± SEM. *p* < 0.05 means the statistical difference. * *p* < 0.05; ** *p* < 0.01; *** *p* < 0.001 and ns, not significant.

To explore the underlying mechanism of GUANKE’s antiviral activity, we investigated its effects on the type I interferon pathway. As shown in Figure 4A, A549 cells were either pretreated with GUANKE or not for 24 h, then infected with RSV (MOI = 0.1) for another 24 h. The IFN-β gene expression was quantitatively analyzed by RT-qPCR. In the GUANKE-pretreated group, IFN-β expression was significantly upregulated compared to the non-pretreated group. Furthermore, ELISA results for IFN-β content in the supernatant of A549 cells at 48 h post-infection (Figure 4B) demonstrated that GUANKE pretreatment led to a marked increase in IFN-β secretion compared to non-pretreated cells (66.99 ± 4.201 vs. 118.8 ± 15.79). To evaluate the antiviral effect of GUANKE in cells deficient in the interferon response, the viral load in IFNAR^−/−^ cells (Figure 4C) and Vero cells (an interferon-deficient cell line) (Figure 4D) was measured by RT-qPCR. GUANKE-pretreated cells showed a significant decrease in viral load compared to non-pretreated cells. Notably, consistent results were observed in influenza A virus (IAV) infection models, where GUANKE treatment significantly suppressed IAV M1 protein expression and upregulated IFN-β expression in vitro (Figure S2A,B), confirming the universal role of the type I interferon pathway in GUANKE-mediated antiviral activity against respiratory viruses.

### 3.5. Lactiplantibacillus plantarum GUANKE Promotes IFN-β Expression in the Early Phase

Figure 4E present the time-course changes in IFN-β expression and secretion in A549 cells. IFN-β expression were significantly elevated in GUANKE-treated cells compared to untreated cells at the indicated time points. In addition, the effects of GUANKE on the IFN-β response in vivo were assessed. IFN-β expression in lung tissue was quantified by RT-qPCR (Figure 4F) and IFN-β content in lung homogenates was measured by ELISA at day 1 post-infection (Figure 4G). GUANKE-treated mice exhibited significantly higher IFN-β expression and productions in lung tissue compared to untreated mice. These results collectively suggest that GUANKE exerts antiviral effects by enhancing type I interferon response.

### 3.6. Lactiplantibacillus plantarum GUANKE Exerts Antiviral Effects Against RSV by Activating Intracellular Signaling Pathway STING-TBK1-IRF3-IFN

**Figure 5:**
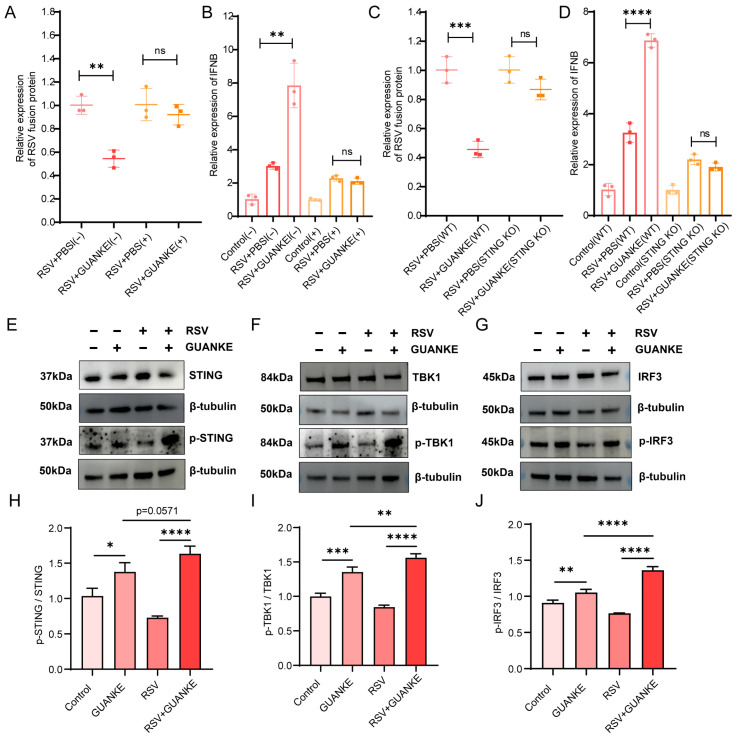
GUANKE activates phosphorylation of STING, TBK1, and IRF3 to modulate antiviral signaling pathways. (**A**) Viral load was quantified by RT-qPCR in cells treated with Cytochalasin D. (**B**) IFN-β gene expression levels were determined by RT-qPCR in cells treated with Cytochalasin D. (**C**) Viral load was quantified by RT-qPCR in STING knockout cells. (**D**) IFN-β gene expression levels were determined by RT-qPCR in STING knockout cells. (**E**–**G**) Western blot analysis of the phosphorylation levels of specific antiviral signaling proteins (**E**) STING, (**F**) TBK1, and (**G**) IRF3. (**H**–**J**) Quantitative analysis of the phosphorylation levels of specific antiviral signaling proteins (**H**) STING, (**I**) TBK1, and (**J**) IRF3 normalized to total protein levels from Western blot results. Data are presented as mean ± SEM. *p* < 0.05 means the statistical difference. * *p* < 0.05; ** *p* < 0.01; *** *p* < 0.001; **** *p* < 0.0001 and ns, not significant.

To elucidate the signaling pathways through which GUANKE mediates its antiviral activity against RSV, we designed and carried out a series of experimental investigations. As shown in Figure 5A, cells were treated with Cytochalasin D to inhibit endocytosis and then infected with RSV. The viral load was quantified by RT-qPCR. Compared with untreated cells, Cytochalasin D-treated cells exhibited the disappearance of the protective effect of GUANKE, indicating that GUANKE-mediated antiviral effects may involve endocytosis-related signaling pathways. RT-qPCR (Figure 5B) analyses of IFN-β gene expression in Cytochalasin D-treated cells revealed that GUANKE-induced IFN-β responses were partially suppressed by Cytochalasin D treatment (RSV + PBS[−] vs. RSV + GUANKE[−], *p* < 0.01, RSV + PBS[+] vs. RSV + GUANKE[+], *p* > 0.05), further supporting the involvement of endocytosis-mediated signaling. To investigate the specific signaling proteins involved, we utilized an A549 cell line with STING knockout (STING KO) to evaluate the significance of STING in GUANKE-mediated antiviral activity. The results demonstrated that in the STING KO cell line experiments (Figure 5C,D), GUANKE failed to reduce viral load (RSV + PBS [WT] vs. RSV + GUANKE [WT], *p* < 0.001; RSV + PBS [STING KO] vs. RSV + GUANKE [STING KO], *p* > 0.05), and did not induce an increase in IFN-β expression levels (RSV + PBS [WT] vs. RSV + GUANKE [WT], *p* < 0.0001; RSV + PBS [STING KO] vs. RSV + GUANKE [STING KO], *p* > 0.05). These findings suggest that STING is essential for GUANKE’s ability to suppress RSV replication. Subsequently, Western blot analysis was performed to assess the phosphorylation levels of STING (Figure 5E), TBK1 (Figure 5F), and IRF3 (Figure 5G). GUANKE-treated cells showed increased phosphorylation of these proteins compared to untreated cells. Quantitative analysis of the phosphorylation levels normalized to total protein levels (Figure 5H–J) confirmed that GUANKE-induced phosphorylation of STING, TBK1, and IRF3 was significantly enhanced. These results collectively demonstrate that GUANKE exerts antiviral effects against RSV by activating the phosphorylation of STING, TBK1, and IRF3 in intracellular signaling pathways.

## 4. Discussion

The immunomodulatory and antiviral activities of GUANKE have been established in our previous studies. In the present work, we investigated the impact of GUANKE on RSV infection and its underlying mechanisms from an interferon-related perspective. We observed a reduction in viral load both in vitro and in vivo following GUANKE treatment. Furthermore, the GUANKE intervention group exhibited significantly reduced lung tissue pathological damage and decreased expression of inflammatory cytokines (IL-1β, IL-6, MCP-1, TNF-α). GUANKE augmented the early type I interferon response and activated the STING-TBK1-IRF3-IFN signaling pathway.

It is well established that interferons play a pivotal role in suppressing viral replication and restricting viral infection. During the development and evolution of viruses, many viruses have demonstrated inhibitory properties against interferons, specifically manifested as interferon delay phenomena, especially in respiratory viruses such as coronaviruses, influenza viruses, and RSVS. Experiments show that SARS-CoV-2 infection delays the production of IFN. Compared with poly(I:C) and SeV, the lag phenomenon of SARS-CoV-2 interferon leads to a large amount of viral replication [47]. The results resulting from different interferon intervention durations are different [48]. The survival rate of animal models with viral infection caused by late administration of interferon was greatly reduced [49]. Similarly, at the population level, early intervention with interferon improves the survival rate, while late intervention reduces it [50]. Therefore, the temporal modulation of the interferon response serves as a pivotal factor in mediating antiviral immunity. Accumulating research evidence has demonstrated that following pretreatment with interferon-α/β (IFN-α/β), the replication of influenza viruses will be significantly reduced [51]. This study found that after pre-intervention with *Lactiplantibacillus plantarum* GUANKE rather than post-infection intervention, not only the expression of interferon IFN-β could be increased, but also the interferon phase could be advanced. This might be an important reason why GUANKE has a good antiviral phenotype. GUANKE has a similar antiviral effect to interferon pretreatment, which is consistent with the above-mentioned interferon timing regulation theory.

The microbial flora is a huge treasure trove with a rich variety. Different probiotics have different properties, and their mechanisms are also different. At present, the research hotspots on the antiviral effect of probiotics focus on the overall regulatory role of the intestinal flora, emphasizing the regulation of local immune responses by changing the microecology of the flora, such as the related research on the “gut-X-axis” [52,53]. Of course, the effects of probiotics can also be non-gut microbiota-mediated. They have direct antiviral effects, such as some studies on vaginal viruses and enteroviruses [54,55,56]. In the field of probiotic antiviral research, probiotics can change the levels of certain metabolites in the body. Relevant studies have been quite extensive. For example, probiotics increase the level of short-chain fatty acids in the intestinal tract to exert antiviral effects [38,57]. Some studies have also demonstrated that probiotics can activate the interferon pathway through TLR receptors. For instance, LGG can up-regulate the expression of TLR3 in intestinal epithelial cells, enhance the recognition of viral RNA, and promote the secretion of IFN-α/β [58]. In the lung tissues of mice given *Limosilactobacillus reuteri* KBL346, the symptoms caused by the virus were alleviated, and the levels of inflammatory factors and the expression levels of Toll-like receptor 2 were decreased [59]. However, at present, there are relatively few studies on the activation of intracellular receptors by probiotics. Generally, we believe that probiotics, as bacterial cells, are relatively large in size and usually do not internalize into host cells, which means they typically do not affect intracellular receptors. This might be one of the reasons for the relatively small amount of research. In a probiotic screening work, experiments demonstrated the influence of probiotics on intracellular signals [60]. During the study of *Lactiplantibacillus plantarum*’s resistance to rotavirus PoRV, it was found that *Lactiplantibacillus plantarum* could activate the phosphorylation of STING protein in porcine epithelial cell models and animal models [61]. This is the result of local antiviral effects of probiotics in intestinal tissues, and this study focuses on the distal regulatory effect through the gut–lung axis. This study found that *Lactiplantibacillus plantarum* GUANKE could activate the intracellular signaling STING-TBK1-IRF3-IFN pathway when antagonizing RSV. These results all indicate that we should not ignore the impact of probiotics on the internal signals of host cells.

This study has achieved a certain experimental basis in evaluating the protective effect of *Lactiplantibacillus plantarum* GUANKE on RSV infection, but there are still some limitations that cannot be ignored. Firstly, although in vitro cell experiments and animal models have provided evidence for the research, there are obvious physiological and immune differences from the human body, which may affect the extrapolation of the research results. Preclinical safety evaluations are needed, such as the long-term impact on immune homeostasis. Secondly, the mechanism of action of GUANKE still awaits in-depth exploration. Although research suggests that probiotics may exert their effects by regulating the host’s immune response, probiotics are active organisms with complex components, and the specific components that exert their effects remain unclear. For example, GUANKE may remotely regulate the host immune system through certain bacterial components or the metabolites it generates, thereby inhibiting viral replication or promoting viral clearance. Therefore, future research should focus on analyzing the mechanism of action of the specific components of *Lactiplantibacillus plantarum* GUANKE in the anti-RSV process, clarifying its target points, and providing a theoretical basis for the development of more targeted antiviral strategies.

Overall, we found *Lactiplantibacillus plantarum* have broad application prospects as preventive agents and auxiliary treatment methods for RSV, which provides a potential non-pharmaceutical intervention strategy for high-risk groups, especially showing positive significance in public health prevention and control [62,63,64], such as the elderly, pregnant women and children. With the deepening of basic research and the accumulation of clinical evidence, probiotics are expected to become an important part of the management of respiratory viral infections, providing new solutions to alleviate the burden brought by RSV. Furthermore, the related research on the combination of probiotics and drugs has brought us new inspirations [65,66]. We expect probiotics to have a synergistic effect with existing antiviral drugs or immunomodulators, thereby improving the therapeutic effect.

## 5. Conclusions

**Figure 6:**
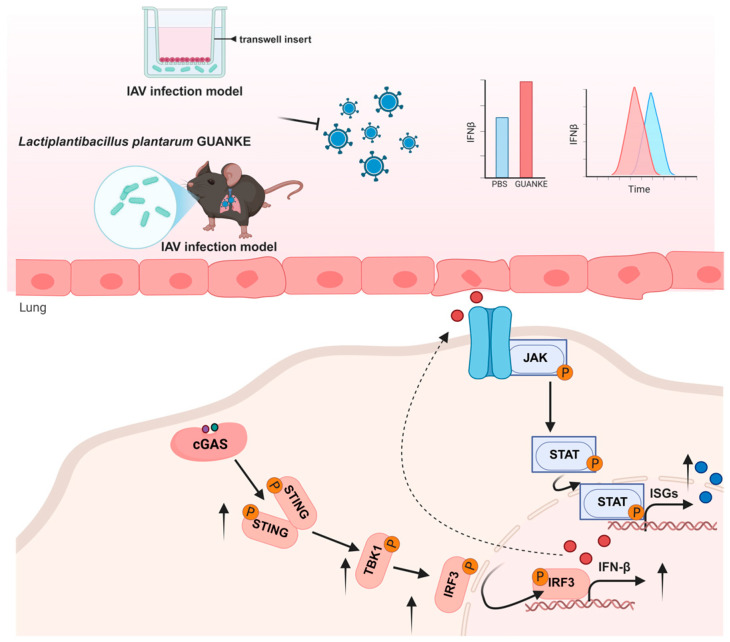
Biological mechanism diagram of the *Lactiplantibacillus plantarum* GUANKE. (Created in Biorender. KunYue. (2025) https://BioRender.com).

To summarize, this study demonstrated the antagonistic effect of *Lactiplantibacillus plantarum* GUANKE on RSV through in vivo animal experiments and in vitro cell models. The potential protective mechanism of *Lactiplantibacillus plantarum* GUANKE in RSV infection was explored. The experiment also observed that the GUANKE intervention group alleviated to a certain extent the pulmonary pathological damage and the down-regulation of inflammatory factors such as IL-6 and IL-1β caused by RSV infection in mice. The mechanism is related to enhancing the early type I interferon response and activating the antiviral pathway. GUANKE intervention increased the expression of interferon and advanced the interferon phase, activated the downstream ISGs expression to block RSV replication, and simultaneously promoted the activation of the intracellular interferon signaling pathway STING-TBK1-IRF3-IFN (Figure 6).

## Figures and Tables

**Table 1:** Primer Sequences for RT-qPCR Amplification.

Primer Name	Sequence
Mouse β-actin	Forward, 5′-TGACGTTGACATCCGTAAAGACC-3′
	Reverse, 5′-CTCAGGAGGAGCAATGATCTTGA-3′
Human β-actin	Forward, 5′-ACTCTTCCAGCCTTCCTTCC-3′
	Reverse, 5′-CGTACAGGTCTTTGCGGATG-3′
Monkey β-actin	Forward, 5′-TGTCCCTGTACGCCTCTG-3′
	Reverse, 5′-ATGTCACGCACGATTTCC-3′
RSV F	Forward, 5′-TAGGAGCCATTGTGTCATGC-3′
	Reverse, 5′-ATCGCACCCGTTAGAAAATG-3′
Mouse *Ifnb1*	Forward, 5′-GCCTTTGCCATCCAAGAGATGC-3′
	Reverse, 5′-ACACTGTCTGCTGGTGGAGTTC-3′
Human *IFNB*	Forward, 5′-GCCGCATTGACCATCTAT-3′
	Reverse, 5′-TAGACATTAGCCAGGAGGTT-3′
*RSAD2*	Forward, 5′-TGGGTGCTTACACCTGCTG-3′
	Reverse, 5′-GAAGTGATAGTTGACGCTGGTT-3′
*ISG15*	Forward, 5′-CGCAGATCACCCAGAAGATCG-3′
	Reverse, 5′-TTCGTCGCATTTGTCCACCA-3′
*IRF7*	Forward, 5′-GCTGGACGTGACCATCATGTA-3′
	Reverse, 5′-GGGCCGTATAGGAACGTGC-3′
*MX1*	Forward, 5′-GTAATGTGGACATCGCCAC-3′
	Reverse, 5′-TCAAGATTCCGATGGTCCTG-3′
*STAT2*	Forward, 5′-CCAGCTTTACTCGCACAGC-3′
	Reverse, 5′-AGCCTTGGAATCATCACTCCC-3′
*IFIT3*	Forward, 5′-TCAGAAGTCTAGTCACTTGGGG-3′
	Reverse, 5′-ACACCTTCGCCCTTTCATTTC-3′
*OASL*	Forward, 5′-CCATTGTGCCTGCCTACAGAG-3′
	Reverse, 5′-CTTCAGCTTAGTTGGCCGATG-3′

## Data Availability

The original contributions presented in this study are included in the article/Supplementary Material. Further inquiries can be directed to the corresponding author.

## Supplementary Materials

The following supporting information can be downloaded at: https://www.mdpi.com/article/10.3390/nu18030399/s1.

## Abbreviations

RSV respiratory syncytial virus, ISG interferon-stimulated genes, RSAD2 Radical S-Adenosyl Methionine Domain Containing 2, TCID_50_ 50% tissue culture infectious dose, dpi day post-infection.
