# Supplementary material for: Lactiplantibacillus plantarum GUANKE Enhances Antiviral Defense Against Respiratory Syncytial Virus Through the STING-TBK1-IRF3-IFN Pathway
Source: Nutrients. 2026 Jan 26;18(3):399. doi: 10.3390/nu18030399 (PMC12898999; doi:10.3390/nu18030399)
Supplement: Supplementary file 1 [file nutrients-18-00399-s001.zip › nutrients-3958308-supplementary.pdf]

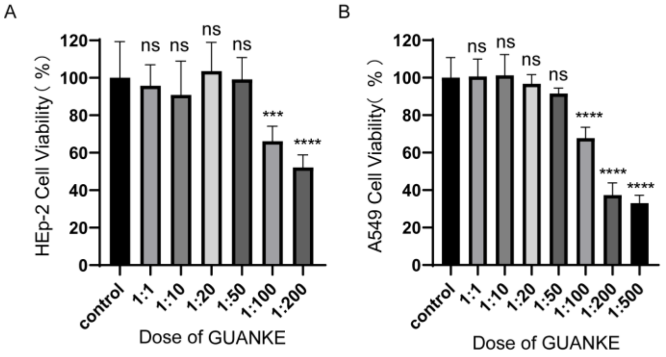

C

HEp-2 Cell Viability

MOI	control	1:1	1:10	1:20	1:50	1:100	1:200
%	100±5.577	95.74±4.592	90.80±8.137	103.6±6.283	99.15±4.770	66.16±3.253	52.12±2.740

D

A549 Cell Viability

MOI	control	1:1	1:10	1:20	1:50	1:100	1:200	1:500
%	100±4.377	100.7±3.780	101.3±4.505	96.79±1.992	91.56±1.190	67.72±2.391	37.30±2.680	33.08±1.710

Figure S1. Viability of HEp-2 and A549 cells treated with different concentrations of GUANKE. (A-B) Cell viability of HEp-2 (A) and A549 (B) cells treated with different doses of GUANKE. (C-D) Quantitative analysis of HEp-2 (C) and A549 (D) cell viability (expressed as percentage of control) following treatment with various doses of GUANKE.

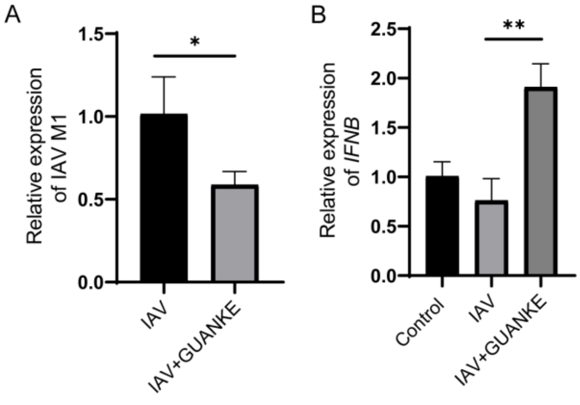

Figure S2. Effects of GUANKE on the expression of influenza A virus (IAV) M1 and IFN-β *in vitro*. (A) Viral load in A549 cells were pretreated with or without GUANKE (MOI=50) was quantified by RT–qPCR. (B) IFN-β expression in A549 cells was quantified by RT-qPCR. A549 cells were pretreated with or without GUANKE for 24 h and then infected with IAV (MOI=0.1) for 24 h.
